# Systematic review and meta-analysis of propranolol in the prevention and treatment of post-traumatic stress disorder

**DOI:** 10.3389/fphar.2025.1545493

**Published:** 2025-01-29

**Authors:** Haipeng Li, Zhengrong Zhang, Shaojie Yang, Guoqi Zhu

**Affiliations:** ^1^ Key Laboratory of Molecular Biology (Brain diseases), Center for Xin’an Medicine and Modernization of Traditional Chinese Medicine of IHM, Anhui University of Chinese Medicine, Hefei, China; ^2^ Acupuncture and Moxibustion Clinical Medical Research Center of Anhui Province, The Second Affiliation Hospital of Anhui University of Chinese Medicine, Hefei, China

**Keywords:** PTSD, propranolol, meta-analysis, systematic review, cardiac dysfunction

## Abstract

**Objective:**

This systematic review and meta-analysis aim to comprehensively evaluate the efficacy of propranolol in the prevention and treatment of post-traumatic stress disorder (PTSD), with a focus on its improvement of core PTSD symptoms.

**Methods:**

A literature search was conducted across multiple databases (including PubMed, Cochrane, Web of Science, and Embase), with the search cutoff date in October 2024. The studies included randomized controlled trials (RCTs) investigating pharmacological treatments for PTSD. PTSD symptoms were assessed using standardized clinical scales, including the Clinician-Administered PTSD Scale (CAPS) and the PTSD Checklist (PCL). The primary outcome was the improvement in PTSD symptoms.

**Results:**

Seven studies met the inclusion criteria for the meta-analysis. The studies showed low heterogeneity, with a chi-squared value of 2.56 (df = 6, p = 0.86) and I^2^ = 0%. The overall effect test indicated significant improvement in PTSD symptoms after propranolol intervention (Z = 2.32, p = 0.02). These findings suggest that propranolol has a statistically significant effect on reducing the severity of PTSD symptoms, with a moderate effect size according to Cohen’s criteria.

**Conclusion:**

This systematic review and meta-analysis provide preliminary evidence supporting the possible role of propranolol in alleviating PTSD symptoms. Future researches are needed to further clarify the therapeutic potential, mechanisms of action, and long-term safety of propranolol in PTSD treatment.

## 1 Introduction

Post-Traumatic Stress Disorder (PTSD) is a psychological condition that often develops after experiencing traumatic events. These events can include natural disasters, serious accidents, warfare, or personal assaults (Boersma-van Dam et al., 2024; Maercker et al., 2022). PTSD is characterized by intrusive memories, hypervigilance, and avoidance behaviors, etc. Additionally, it can lead to negative changes in emotional and cognitive functioning, such as feelings of hopelessness, emotional numbness, and difficulty maintaining close relationships (Alkalame et al., 2024; Compean and Hamner, 2019; Maytles et al., 2024). If left untreated, these symptoms may persist for months or even years, significantly impairing the individual’s daily life (Panayi et al., 2024). Despite the prevalence and severe impact of PTSD, effective prevention and treatment strategies remain a crucial area of research.

Due to the interplay of multiple factors, psychological stress and anxiety are common in individuals with chronic conditions, with the interaction between cardiac dysfunction and PTSD being particularly significant (Bernardi et al., 2020; Roy et al., 2015; Vilchinsky et al., 2017). This condition involves dysregulation of the autonomic nervous system and the hypothalamic-pituitary-adrenal (HPA) axis (Cohen et al., 2020; Seligowski et al., 2022; von Känel et al., 2010), impairing cardiac function and exacerbating stress responses. Chronic inflammation, which is common in both PTSD and cardiovascular disease, may further increase the likelihood of PTSD. The hyperarousal associated with PTSD, manifested by increased heart rate (HR) and blood pressure, may worsen existing cardiac dysfunction (Buckley et al., 2004; Lee et al., 2022). Overall, the combined physical and psychological stress experienced by these patients increases the risk of developing PTSD (Hargrave et al., 2022), emphasizing the need for an integrated approach to managing both cardiac and mental health. Given these overlapping mechanisms, interventions such as β-blockers, specifically propranolol, may help reduce physiological stress responses in PTSD patients with cardiac dysfunction.

Propranolol exerts its effects primarily by blocking β-adrenergic receptors, which alleviates symptoms of hyperarousal (e.g., tachycardia and hypertension) and reduces physiological responses associated with excessive sympathetic nervous system activation, thereby mitigating emotional stress and the impact of trauma (Winzenried et al., 2023). This pharmacological action is believed to interfere with the consolidation of traumatic memories, a hallmark feature of PTSD (AlOkda et al., 2019), by reducing the physiological markers of stress, such as elevated heart rate, that are commonly associated with the disorder.

As one of the potential drug treatment methods for PTSD, the efficacy of propranolol in alleviating post-traumatic stress symptoms, especially high arousal and invasive memory, has been explored in many animal experiments and some clinical studies (Brunet et al., 2018; Burhans et al., 2018; Elsey et al., 2020; Roullet et al., 2021; Thierrée et al., 2020). While some studies have reported positive outcomes, the evidence remains inconclusive, indicating that the effectiveness and optimal intervention protocols for propranolol in PTSD still require further research. Therefore, the aim of this systematic review and meta-analysis is to critically evaluate the existing studies on propranolol intervention for PTSD.

## 2 Methods

We followed the methodology outlined in the Cochrane Handbook for Systematic Reviews of Interventions and completed the PRISMA checklist.

### 2.1 Inclusion and exclusion criteria

Inclusion Criteria: (a) Randomized controlled trials (RCTs), including cluster and crossover designs; (b) Studies investigating the effect of pharmacological interventions after a traumatic event; (c) Comparisons with placebo, pharmacological, or psychological interventions; (d) Participants exposed to traumatic events that may meet the criteria for PTSD according to the Diagnostic and Statistical Manual of Mental Disorders (DSM) A criteria; (e) Use of one or more validated clinician-administered or self-reported outcome measures to assess PTSD symptoms.

There were no restrictions regarding the severity of PTSD symptoms or the type of traumatic event, and no sample size restrictions were applied. Only studies published in English were included.

### 2.2 Search strategy and selection criteria

All RCTs that reported on the use of propranolol in the prevention or treatment of PTSD were identified and reviewed, yielding 521 relevant papers. The initial search strategy included databases such as PubMed, Web of Science, Embase, and the Cochrane Randomized Trials Database, with no time limit on the search date. PTSD-related terms were combined with terms related to pharmacological treatments (using both Medical Subject Headings [MeSH] and text words).

All titles and abstracts were screened by two independent reviewers, followed by a thorough discussion to ensure agreement. Full-text articles were obtained for all potentially relevant studies. If the full text was unavailable, the corresponding authors were contacted to request the manuscript. To determine whether the studies met the inclusion criteria, the full texts were independently reviewed by two researchers.

### 2.3 Data extraction

Data were independently extracted by two researchers using a standardized data extraction form. If additional information was required, the authors of the studies were contacted. The extracted data included basic demographic details, specifics of the interventions used, as well as the primary outcomes related to the severity and incidence of PTSD symptoms.

### 2.4 Risk of bias assessment

The Cochrane Collaboration’s tool for assessing the risk of bias in randomized trials was used for each identified study. This tool evaluates the likelihood of bias in randomized trials based on factors including: Adequacy of the allocation sequence generation, Acceptability of allocation concealment, Adequacy of participant and personnel blinding, and the degree of incomplete outcome data.

Risk of bias was independently assessed by two reviewers, with any disagreements resolved through discussion. These ratings were then considered in a GRADE assessment (which evaluates the quality of evidence to make recommendations for clinical practice) for each outcome.

### 2.5 Data synthesis

The primary outcome of the meta-analysis was the reduction in PTSD symptoms following pharmacological treatment after a traumatic event. A fixed-effects model was used for the analysis. Additionally, PTSD incidence 3–6 months after the traumatic event was considered. For the success rate of propranolol intervention in PTSD, relevant confidence intervals were calculated. Statistical heterogeneity was measured using the I^2^ statistic for results involving multiple studies. Subgroup analyses were not performed due to the limited number of studies addressing individual outcomes. All analyses were conducted using Review Manager 5.4.1 software from the Cochrane Collaboration.

## 3 Results

### 3.1 Study characteristics

**FIGURE 1:**
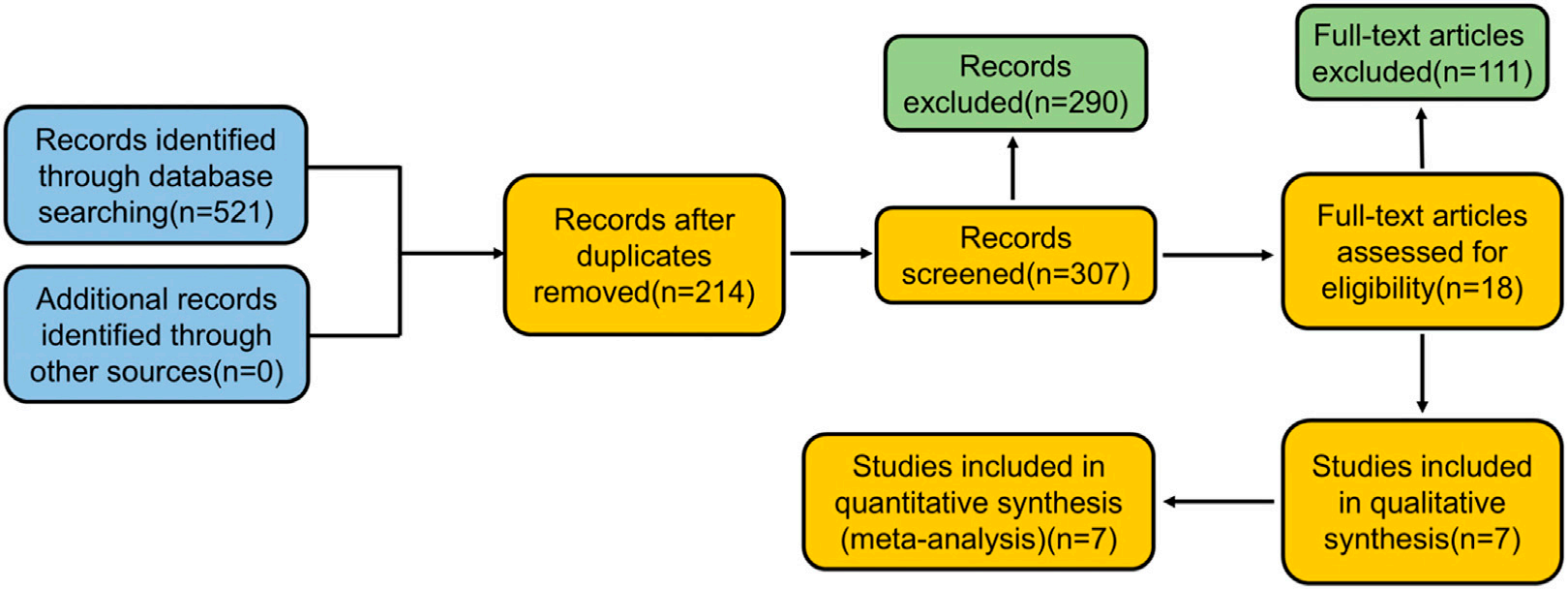
Flow diagram: study selection.

A total of 521 papers were identified from the search. The full texts of these 521 studies were reviewed, and 307 articles met the inclusion criteria. During the review process, 514 studies were excluded, as shown in Figure 1. The systematic review ultimately included seven studies, involving a total of 251 participants. Data extracted from these seven selected studies are summarized in Table 1.

**TABLE 1:** Overview of the seven studies included in the meta-analysis.

Study	Country	Age	Trauma sample and assessment criteria	Timing of administration post-trauma	Comparator	Final sample size	PTSD outcomes	Propranolol dosage
Hoge	United States	18–65	Emergency department patients (DSM-IV)	4 h post-trauma	Placebo	34	CAPS	240 mg of propranolol orally once a day for 19 days
Brunet 2018	Canada	18–65	Patients with PTSD for at least 6 months (DSM-IV)	Propranolol treatment prior to reactivation	Placebo	60	PCL-S CAPS	1.67 mg of propranolol orally once a week for 6 weeks
Roullet	France	18–65	Patients with PTSD for at least 3 months (DSM-IV)	Administered 90 min prior to memory reactivation	Placebo	50	PCL-S SCID	1.67 mg of propranolol orally once a week for 6 weeks
Brunet 2021	Nepal	25–65	Patients who have experienced traumatic events (DSM-IV)	Propranolol administered during trauma reactivation	Paroxetine	34	PCL HSCL-25 MINI 5.0.0	1 mg/kg of propranolol orally once a week for 6 weeks
Pitman	United States	None	Emergency Department Patients (DSM-IV)	Taken as soon as possible after the traumatic event	Placebo	26	CAPS	40 mg of propranolol orally 4 times a day for 10 days
Vaiva	France	21–30	2–20 h post-trauma (DSM-IV)	Administered immediately after a 20-min baseline heart rate recording period	No medication	19	The Peritraumatic Distress Inventory	40 mg of propranolol orally 3 times a day for 7 days

Given that physiological symptoms are an indirect measure of PTSD severity, studies evaluating the effects of propranolol on memory reactivation and PTSD symptoms were analyzed separately.

Pitman et al. (2002) conducted a study with 41 emergency department (ED) patients who experienced traumatic events that met DSM-IV PTSD criteria A.1 (stressors) and A.2 (responses), with a HR of at least 80 beats per minute (bpm) and no severe physical injuries such as congestive heart failure, heart conduction block, or a history of bronchial asthma. These patients were deemed capable of understanding the purpose and procedures of the study and provided written informed consent. Within 6 h of the traumatic event, patients received an initial dose of 40 mg propranolol or a placebo. After approximately 1 hour, patients were allowed to leave the ED and were instructed to continue taking the medication four times daily for 10 days, followed by a 9-day tapering period. After excluding outliers a month later, the PTSD incidence rate in the placebo group was 30% (6/20), while in the propranolol group, it was 10% (1/10). Three months later, 11% (1/11) of the propranolol group and 13% (2/15) of the placebo group met the DSM-IV criteria for chronic PTSD. Additionally, no significant abnormal physiological responses were observed in either group.

Vaiva et al. (2003) recruited trauma victims aged 21–30 years from a French hospital ED, all of whom had developed tachycardia (resting HR ≥ 90 bpm after 20 min) following a traffic accident or personal assault. Exclusion criteria included loss of consciousness, severe physical or brain injury, cardiovascular disease, or a history of PTSD. The severity of the trauma was assessed using a pre-trauma distress checklist, trauma scores, PTSD treatment outcomes scale, and DSM-IV criteria, with PTSD symptoms and trauma history evaluated through the International Neuropsychiatric Interview. Participants provided informed consent prior to receiving 40 mg propranolol treatment, starting immediately after baseline heart rate recordings, followed by a 7-day treatment period and a gradual tapering phase. Of the 54 eligible trauma victims, 31 were potential participants, 23 met the resting heart rate criteria, and 11 received propranolol. Two months after the trauma, the PTSD incidence in the propranolol group (1/11) was lower than in the non-medication group (3/8), with significantly lower symptom scores in the propranolol group. Treatment adherence was good, with minimal side effects, although two patients abruptly discontinued propranolol, one of whom experienced severe anxiety.

Stein et al. (2007) conducted a randomized, double-blind, placebo-controlled trial to evaluate the efficacy of pharmacological interventions in trauma patients admitted to a surgical trauma center. The study initially screened 5,062 individuals, ultimately enrolling 48 participants who were randomly assigned to one of three groups: propranolol (n = 17), gabapentin (n = 14), or placebo (n = 17). The primary objective was to assess the impact of propranolol on PTSD following a traumatic injury. Treatment with propranolol was initiated within 48 h of injury and continued for a total of 14 days. The regimen was structured in three phases: an escalation phase lasting 2 days, an acute phase lasting 8 days, and a tapering phase lasting 4 days. The starting dose of propranolol was 20 mg, administered three times daily, with a subsequent increase to 40 mg after 2 days. PTSD symptoms were assessed using the Post-Traumatic Stress Checklist-Civilian Version (PCL-C) at three time points: 1, 4, and 8 months post-injury. The study results indicated a significant reduction in PCL-C scores over time for the propranolol group, suggesting that early administration of propranolol may be effective in mitigating PTSD symptoms in trauma patients. The trial’s findings support the hypothesis that pharmacological intervention, particularly with propranolol, may offer a potential therapeutic approach for preventing the onset of PTSD following traumatic events. However, further research is needed to confirm these results and explore the long-term benefits and safety of propranolol in this context.

Hoge et al. (2012) investigated the effects of propranolol on PTSD in individuals exposed to traumatic events. Participants, aged 18–65 years, were recruited from the ED between September 2004 and May 2008. To qualify for the study, individuals needed to meet DSM-IV criteria for PTSD and experience a traumatic event within 12 h of receiving the study drug. Exclusion criteria included contraindications to propranolol, current substance abuse, certain mental health conditions, pregnancy, and suicidal tendencies. After screening and obtaining informed consent, participants were randomly assigned to receive either 40 mg of short-acting propranolol, 60 mg of long-acting propranolol, or a placebo. Medication adherence was carefully monitored using participant logs, pill counts, and electronic tracking devices. PTSD symptoms were assessed using the Clinician-Administered PTSD Scale (CAPS) and the Structured Clinical Interview for DSM-IV Dissociative Disorders (SCID). The study found that, in *post hoc* subgroup analyses, participants with high drug adherence showed significantly reduced physiological reactivity during script-driven imagery at the 5-week post-trauma assessment, with the propranolol group exhibiting lower reactivity compared to the placebo group. These findings suggest that propranolol may have potential as an early intervention to reduce physiological responses to trauma-related stimuli, potentially mitigating the development of PTSD in individuals with high adherence to the treatment regimen.

Brunet et al. (2018) conducted a 6-week, double-blind, placebo-controlled, randomized clinical trial to investigate the effects of pre-reactivation propranolol therapy on PTSD symptoms. Sixty-one participants (ages 18–65) were randomized to receive either propranolol or placebo, with up to 6 treatments. Participants were selected based on specific inclusion criteria, including a PTSD diagnosis and certain health conditions, while exclusion criteria included cardiovascular diseases or current substance dependence. The combination of short-acting and long-acting propranolol was administered, and vital signs were monitored before and after treatment. Six participants reported side effects that led to their exclusion from the study. Treatment effects were measured using the CAPS and PTSD Checklist-Specific (PCL-S) before and after treatment. Results showed that PTSD symptoms significantly improved in the propranolol group compared to the placebo group, regardless of whether assessed by CAPS or PCL-S. This improvement was consistent in both intention-to-treat and per-protocol analyses. At the 6-month follow-up, the propranolol group had lower PTSD scores compared to the placebo group. These findings suggest that pre-trauma activation with propranolol may be a potential PTSD prevention strategy. However, while the study indicates that propranolol might impair trauma memory consolidation, it did not directly confirm this mechanism, and further research is needed to explore this hypothesis, such as testing the timing of propranolol administration relative to memory reactivation.

Additionally, Brunet et al. (2021) conducted a study in Nepal between 2013 and 2014 to assess the efficacy of PTSD treatment methods, including propranolol combined with reconsolidation therapy (RT) and paroxetine (SSRI). Participants aged 25–65 who agreed to participate had experienced a traumatic event that met DSM-IV-TR PTSD criteria (symptoms lasting >6 months). Exclusion criteria included low heart rate, low blood pressure, and active suicidal ideation. The study used a 1:1 design and treated the RT group with 1 mg/kg propranolol weekly for 6 weeks. Paroxetine was administered at a dose of 20 mg/day for 13 weeks, followed by 10 mg/day for an additional 13 weeks. Participants took the medication at home, with transportation costs reimbursed to ensure adherence to the treatment plan. PTSD symptoms were assessed using the PCL, and psychiatric comorbidities were assessed using the Hopkins Symptom Checklist (HSCL-25). In total, 40 participants (87%) completed 6 weeks of treatment, and 34 (74%) completed the study per protocol. At the 7th week, both treatment groups showed significant improvements, and both treatments showed sustained improvement at the 13th and 26th weeks, with no participant showing PTSD severity scores exceeding the cutoff at the 26-week follow-up. Although there was gender imbalance in the study, with more female participants, the main analysis using repeated measures mixed models showed significant treatment effects over time in both groups (Brunet et al., 2021).

Roullet et al. (2021) conducted a study to evaluate the efficacy of propranolol, a beta-blocker, in blocking the reconsolidation of trauma memories in individuals with PTSD. In this double-blind, placebo-controlled trial, 66 adults diagnosed with chronic PTSD were randomized to receive either propranolol or a placebo, with weekly treatments administered over a 6-week period. As part of the treatment protocol, participants’ trauma memories were reactivated after the medication was administered. Both groups experienced significant reductions in PTSD symptoms and comorbid depression during the treatment phase. However, there was no significant difference in symptom reduction between the propranolol and placebo groups throughout the treatment period. At the 3-month follow-up, PTSD and depression scores continued to improve in the propranolol group, with scores on the PCL-S remaining above 65, suggesting a sustained therapeutic effect. In contrast, the placebo group’s PTSD and depression scores worsened over time. These results indicate that while propranolol did not show immediate superiority over placebo in symptom reduction during treatment, it may offer long-term benefits in preventing symptom relapse in individuals with chronic PTSD.

**FIGURE 2:**
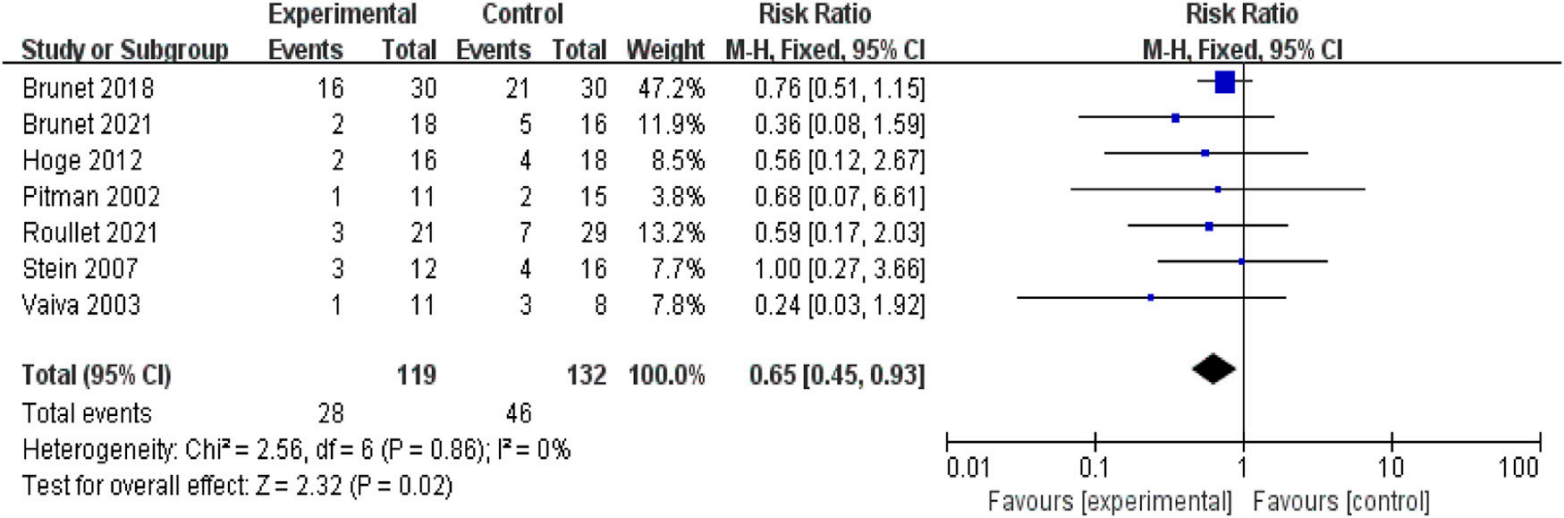
Meta-analysis: comparison between propranolol and comparator in the prevention and treatment of PTSD.

Using data from the seven studies above, a meta-analysis was performed, enrolling 251 participants in total. Figure 2 shows the forest plot generated using a fixed-effects model for the meta-analysis. Each study provides the relative risk and 95% confidence interval (CI). The relative risk estimate for the efficacy of propranolol in preventing or treating PTSD is 0.65 (95% CI: 0.45–0.93).

### 3.2 Risk of bias assessment

**FIGURE 3:**
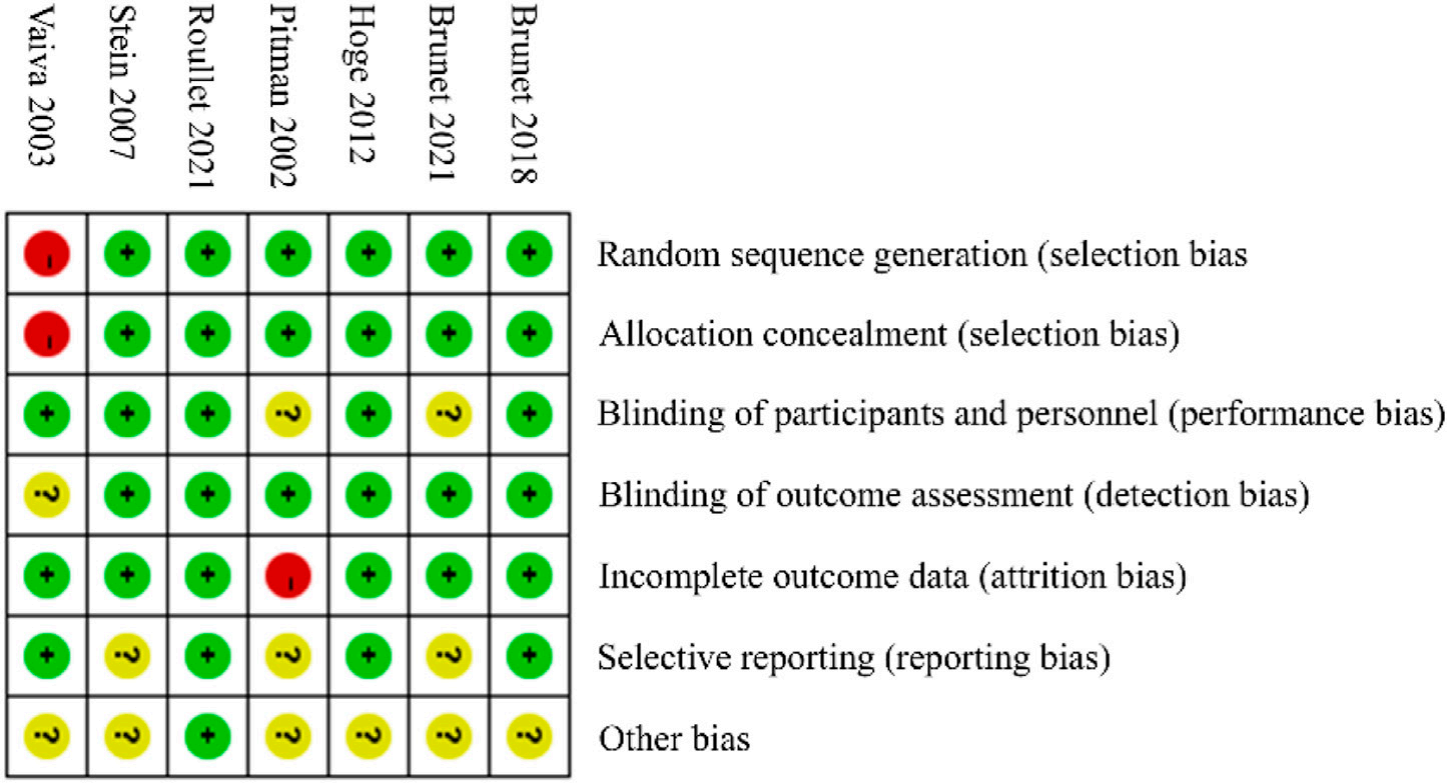
Summary of risk of bias assessment.

The summary of the risk of bias assessment is presented in Figure 3. Specifically, among the 7 clinical trials included in this analysis, the study by Vaiva et al. reported an early investigation of PTSD patients. This study showed a slight risk of bias in the methodology section, which may have influenced the overall results.

**FIGURE 4:**
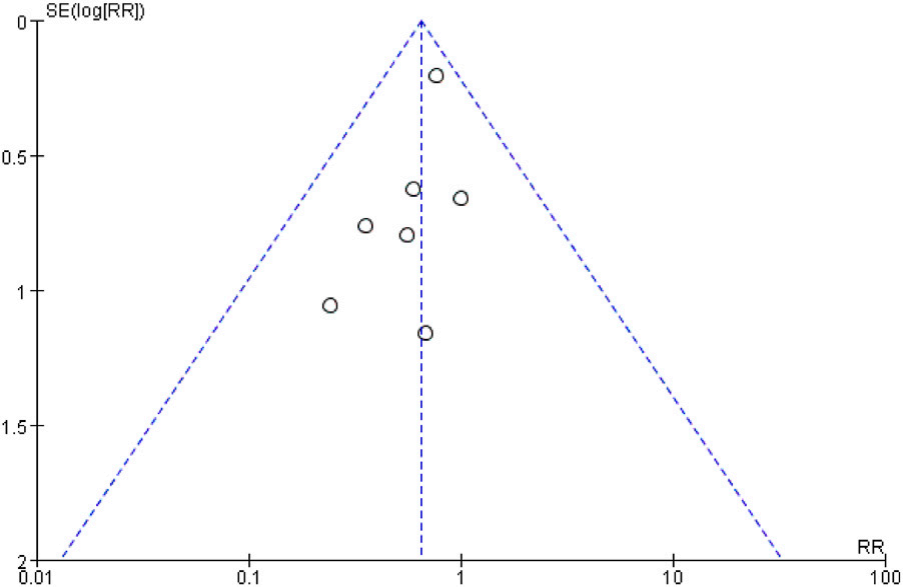
Funnel plot of publication bias.

Regarding publication bias, Figure 4 presents a funnel plot. Table 2 shows the results of the Egger and Begg tests, which indicate no significant publication bias (p > 0.05).

**TABLE 2:** Egger and begg tests for the 7 studies in the meta-analysis.

Publication bias test			
Egger’s regression-based test			
beta1	−0.608	Standard error (SE)	0.355
t-value	−1.712	Degrees of freedom (df)	5
p-value	0.147 > 0.05		
Begg’s test for rank correlation			
Kendall’s score	−9.000	Standard error (SE)	6.658
z-value	−1.352	p-value	0.176 > 0.05

## 4 Discussion

Compared to previous meta-analyses, our study reached differentiated conclusions after including new literature. This discrepancy may be due to the disproportionately high weighting of Brunet’s sample. Additionally, a slight conflict between the findings of Pascal Roullet and Alain Brunet may relate to placebo effects in Brunet’s study, where the PCL-S scores in the placebo group showed improvements comparable to those in the propranolol group (Roullet et al., 2021; Brunet et al., 2018). These improvements could be linked to the six-session exposure protocol and the completion of seven PCL-S assessments by participants. Since exposure therapy is a recognized PTSD treatment, such repeated exposure procedures may influence the outcomes. Despite this, these results suggest that propranolol may have a role in treating PTSD, but further research is needed to clarify its potential clinical benefits and indications.

Our findings highlight propranolol’s potential in alleviating physiological symptoms of PTSD, such as hyperarousal and intrusive memories. However, PTSD is a complex disorder involving not only physiological symptoms but also emotional and cognitive symptoms, such as avoidance behaviors and negative mood states. Propranolol may not effectively address these diverse core symptoms. Previous studies have often focused on physiological symptoms, as measured by scales like CAPS and PCL, while failing to explore other critical symptoms, particularly sleep disturbances and avoidance behaviors—key components of PTSD pathophysiology (McFall et al., 1990; Taylor et al., 1981).

Sleep disturbances affect over 90% of PTSD patients (Colvonen et al., 2019). Although propranolol may mitigate hyperarousal by modulating physiological stress responses, its effects on sleep quality and psychological symptoms remain unclear. By contrast, prazosin is considered a more promising medication for PTSD treatment (Hendrickson et al., 2021; Lappas et al., 2024), particularly because it effectively addresses both sleep disturbances and other core symptoms, including hyperarousal and intrusive symptoms (Keeshin et al., 2017; Ressler et al., 2022). Meta-analyses of prazosin interventions for PTSD often utilize diverse statistical approaches, including differences in sleep quality endpoints, nightmare severity, and Surface Under the Cumulative Ranking Curve as statistical indicators (Huang et al., 2024; Khachatryan et al., 2016). Based on these studies, future research should explore potential pharmacological agents for alleviating PTSD symptoms using more comprehensive study designs and robust statistical methods. Additionally, prazosin’s targeting of the noradrenergic system provides broader therapeutic effects on hyperarousal and intrusive symptoms (De Berardis et al., 2015; Strawn and Geracioti, 2008; van Liempt, 2012), further underscoring the critical role of the noradrenergic system in PTSD pathogenesis and maintenance.

Similarly, propranolol, as a noradrenaline inhibitor, shows certain potential. However, current research on its use for PTSD treatment remains in its early stages. Future studies should continue to explore the differences between propranolol and prazosin in terms of efficacy, mechanisms of action, and indications. Given prazosin’s promising clinical outlook, future propranolol research could benefit from adopting similar study designs, including larger sample sizes, stricter inclusion criteria, and thorough monitoring of treatment adherence. This approach may facilitate the exploration of new indications for propranolol.

## 5 Conclusion

The data analyzed in this study suggest that propranolol may have certain benefits for alleviating PTSD symptoms, but the evidence is inadequate to establish it as a reliable and broadly applicable treatment for PTSD. While the diversity in study locations and populations adds some value, it does not imply that positive findings from localized studies could be generalized to all populations. Variations in study design, patient characteristics, and treatment protocols further limit our ability to draw definitive conclusions regarding propranolol’s clinical efficacy.

## Data Availability

The original contributions presented in the study are included in the article/supplementary material, further inquiries can be directed to the corresponding authors.
